# Opening wedge high tibial osteotomy using patient‐specific instruments and custom‐made plates results in accurate correction and improved clinical outcome measures at 1‐year follow‐up

**DOI:** 10.1002/jeo2.70340

**Published:** 2025-07-07

**Authors:** Lukas Jud, Matthias Biner, Lazaros Vlachopoulos, Bastian Sigrist, Sandro F. Fucentese

**Affiliations:** ^1^ Department of Orthopedics Balgrist University Hospital, University of Zurich Zurich Switzerland; ^2^ Research in Orthopedic Computer Science (ROCS) Balgrist University Hospital, University of Zurich Zurich Switzerland

**Keywords:** HTO, osteotomy, personalized implants, PSI, realignment surgery

## Abstract

**Purpose:**

Achieving high accuracy is a primary goal in opening wedge high tibial osteotomy (HTO). However, literature on the combined use of patient‐specific instruments (PSI) and patient‐specific plates (PSP) remains limited. This study investigates the accuracy and clinical results of the combined use of a novel PSI and PSP system.

**Methods:**

All medial open‐wedge HTO using the PSI and PSP system from Medacta (Medacta, Castel San Pietro, Switzerland) between November 2021 and April 2023 at our institution were included. The hip‐knee‐ankle angle (HKA) and the posterior tibial slope (PTS) were measured preoperatively, and at 4.5 months and 1 year postoperatively, to assess surgical accuracy. The knee society score (KSS) was collected preoperatively and at 1 year postoperatively.

**Results:**

Fourteen knees from 14 patients (6 females, 8 males) were included. Accuracy for HKA correction at 1‐year follow‐up was 1.9 ± 1.4°, whereas accuracy for PTS correction was 1.0 ± 0.9°. KSS significantly improved for the clinical score from 57.4 ± 19.4 to 84.1 ± 16.6 (*p* = 0.003) and for the function score from 80.4 ± 18.4 to 93.6 ± 11.5 (*p* = 0.043).

**Conclusion:**

The investigated combined PSI and PSP system shows accurate surgical accuracy for HKA and PTS correction with significant improvement of patient‐reported outcomes at 1‐year follow‐up.

**Level of Evidence:**

Level III.

**Abbreviations**
3D: three‐dimensional
BMI: body‐mass‐index
CT: computed tomography
HKA: hip‐knee‐ankle angle
HTO: high tibial osteotomy
KSS: knee society score
LLR: long‐leg radiographs
PSI: patient‐specific instruments
PSP: patient‐specific plates
PTS: posterior tibial slope
SD: standard deviation
TOKA: Tailored Osteotomy Knee Alignment

## INTRODUCTION

A varus malalignment of the hip‐knee‐ankle angle (HKA) causes an unbalanced load distribution in the knee, potentially resulting in an accelerated degeneration of the medial compartment. In younger patients, joint‐preserving procedures, such as opening wedge high‐tibial osteotomy (HTO), are preferred over knee arthroplasty [19]. In favourable candidates, survivorship of nearly two‐thirds is reported with a satisfaction rate of 97% at 20 years of follow‐up [3].

However, high accuracy in HKA correction is crucial for joint‐preserving procedures, as under‐ or overcorrection is a leading cause of clinical failure [2]. A systematic review showed that over one quarter of patients treated by conventional HTO show an accuracy worse than 3° for HKA correction in eight out of fourteen included studies [1]. To enhance surgical accuracy in HKA correction, navigation methods have been developed, including the use of patient‐specific instruments (PSI) [4, 5, 11, 26]. The beneficial use of PSI has already been proven for different anatomical locations [10, 13, 14]. However, further improvements may be possible by complementing PSI with customized implants in terms of the use of patient‐specific plates (PSP). A recent study showed promising results using PSI combined with PSP, with an accuracy for HKA correction of 2.1° [24]. However, the literature regarding such personalized HTO approaches with the combined use of PSI and PSP is scarce. Hence, this study was performed to investigate the surgical accuracy and clinical results of HTO using a novel PSI and PSP system from Medacta (Medacta, Castel San Pietro, Switzerland). We hypothesize that accurate surgical accuracy with improved patient‐reported outcomes can be achieved at 1‐year follow‐up.

## MATERIAL AND METHODS

### Study population and design

The local ethical committee approved this study (Zurich Cantonal Ethics Commission, BASEC‐Nr. 2023‐00389), and all patients gave their informed consent.

This was a retrospective case series. All patients who underwent opening wedge HTO for medial compartment degeneration or medial compartment overload in case of a varus HKA from November 2021 to April 2023 at our institution, and who were operated by the senior surgeon (SF), were included. Furthermore, the inclusion criteria consisted of the use of the PSI and PSP system from Medacta (Medacta). The used PSP was the MyOstetotomy HTO Custom Plate (Medacta). Exclusion criteria comprised the lack of a postoperative computed tomography (CT) scan and the lack of weight‐bearing long‐leg radiographs (LLR) and lateral knee radiographs taken preoperatively and at 4.5 and 12 months postoperatively.

### Preoperative planning

For preoperative planning, standard weight‐bearing radiographs of the knee and LLR were acquired. Additionally, a CT scan of the extremity was performed, scanning only the regions of interest (i.e., hip‐, knee‐, and ankle‐joint), irrelevant mid‐shaft regions were skipped to reduce radiation exposure to the patient. The CT scan required the inclusion of the hip‐, knee‐, and ankle‐joint for the following appropriate three‐dimensional (3D) surgical planning. Following, CT data was sent to Medacta (Medacta). After reconstruction of 3D triangular surface models [5, 16], HTO was simulated by the Medacta engineers. The height of the opening wedge was calculated and used for building the wedge which was integrated in the MyOstetotomy HTO Custom Plate and fits exactly in the planned osteotomy gap. Following, the proposed 3D planning from Medacta was sent to the treating surgeon, which adapted the planning, taking the conventional radiographs and the LLR into consideration. The osteotomy module of the mediCAD planning software (Hectec GmbH, version V7.0) was used for HTO planning on LLR with a mean HKA correction of 6.6 ± 1.1° (range: 5.0 to 8.0°). After adaption or approval of the 3D planning, the PSI and the PSP were manufactured by Medacta (Medacta).

### Surgical technique

**Figure 1:**
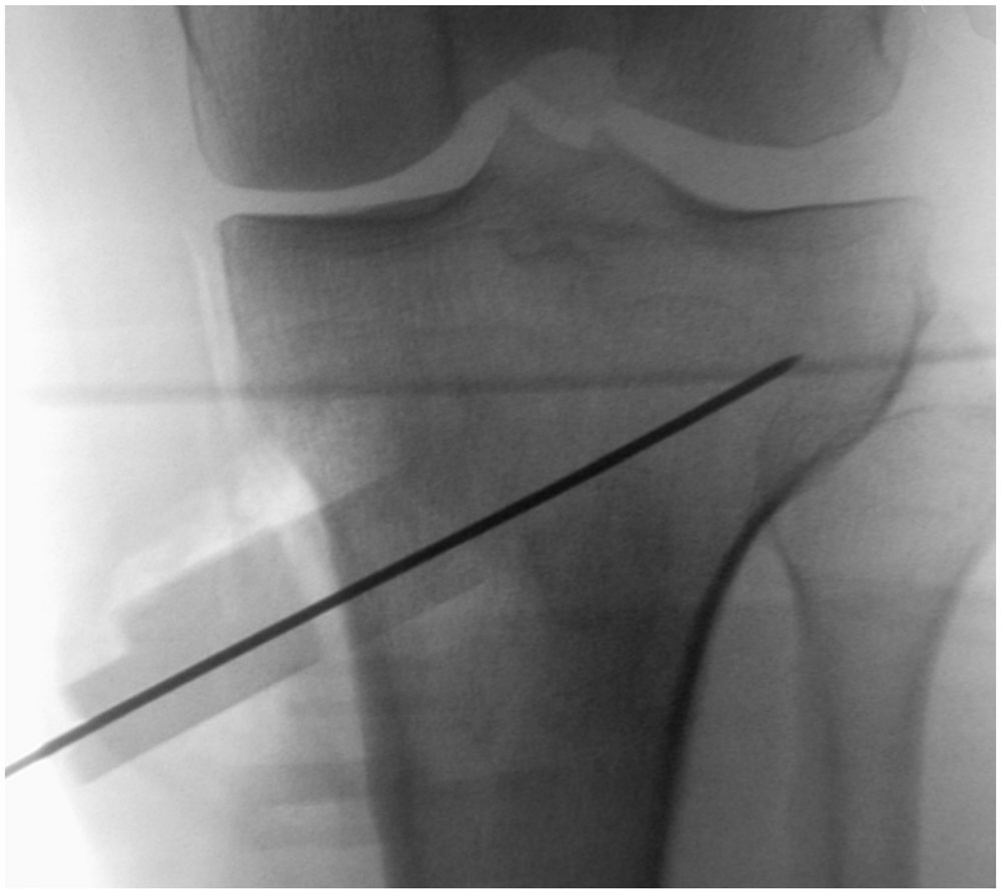
The patient‐specific instrument (PSI) is placed. A K‐wire is drilled through the cutting slot and fluoroscopy is used to control for the position of the PSI.

**Figure 2:**
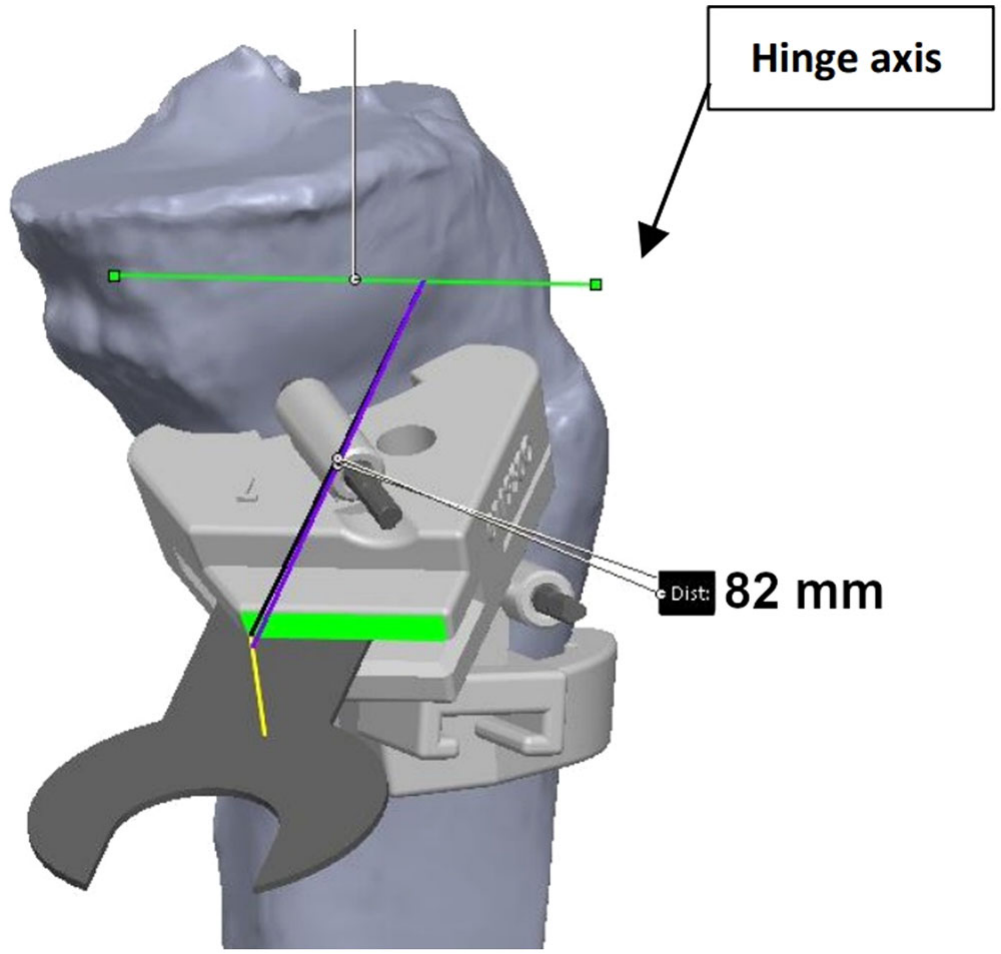
The patient‐specific instrument (PSI) is placed and secured by two 3.2 mm pins (dark grey). The osteotomy is performed through the cutting slot, using a 1 mm saw blade, to the preoperatively defined depth (82 mm in this case).

**Figure 3:**
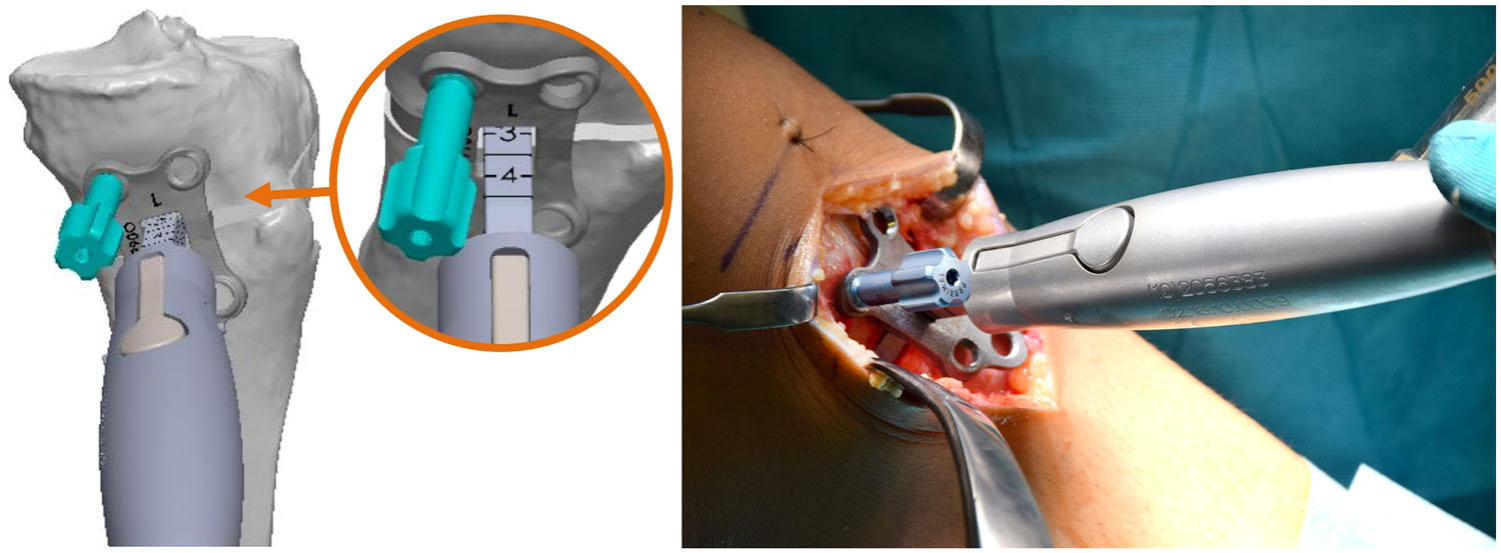
The patient‐specific plate (PSP) is assembled with the wedge tool, which is inserted to the preoperatively defined depth (depth 3 in this case). A drilling guide (turquoise) is positioned in the posterior proximal plate screw hole, which is used to place the plate over the remained 3.2 mm pin. The customized wedge fits exactly inside the osteotomy gap.

**Figure 4:**
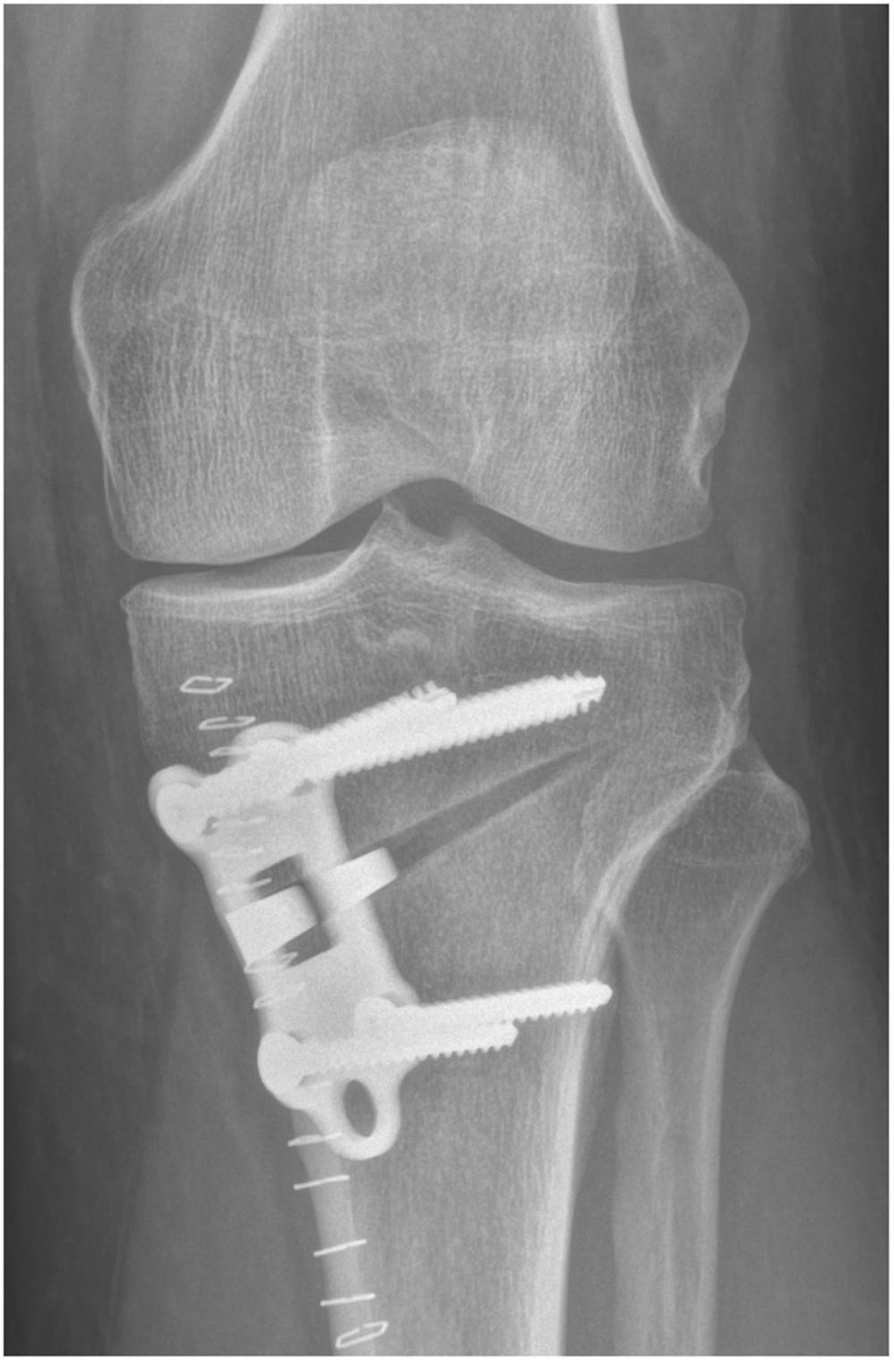
Postoperative radiograph.

**Figure 5:**
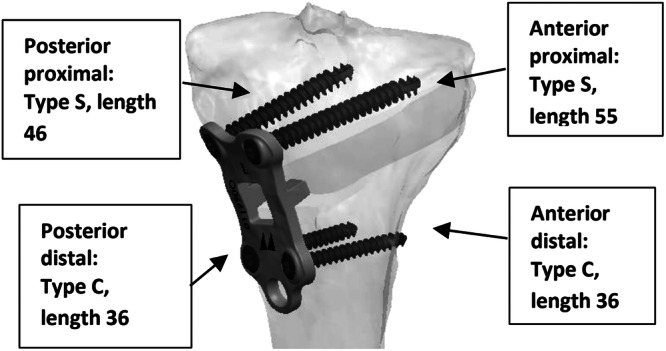
Preoperatively defined screw lengths.

All surgical procedures were performed by the senior surgeon (S. F.). Surgery was performed with the patient in a supine position using general or spinal anaesthesia. A tourniquet was used and opened before closing the wound. An anteromedial approach to the tibial head was performed. A sparing detachment of the medial collateral ligament to the level of the osteotomy was performed and the bone was relieved of the soft tissue for a proper positioning of the PSI. Using fluoroscopy, the position of the PSI was controlled by inserting a 2‐mm k‐wire into the cutting slot (Figure 1). The position of the PSI was secured by inserting one posterior and one anterior 3.2 mm pin into the integrated drill sleeves. Afterwards, the osteotomy was performed through the cutting slot using a 1 mm thick saw blade to the depth, which was defined in the preoperative planning (Figure 2). After removing the anterior 3.2 mm pin, the PSI was removed and the osteotomy was completed behind the tibial tubercle in a proximal directed fashion using the oscillating saw and a chisel. The osteotomy was then carefully opened to the aimed opening‐distance using chisels. Afterwards, the MyOsteotomy HTO Custom Plate (Medacta) was assembled with the wedge tool and a drilling guide in the posterior proximal plate screw hole, which was used to place the plate over the remained 3.2 mm pin (Figure 3). The plate was positioned with the customized wedge inside the osteotomy gap. After controlling for a proper fit of the wedge in the osteotomy gap, screws were consecutively placed using two spongious monocortical screws in the proximal plate and two cortical bicortical screws in the distal plate (Figure 4). Screw length was read from the preoperative 3D planning (Figure 5).

### Radiological assessment

The HKA was measured preoperatively, and at 4.5 and 12 months postoperatively, using weight‐bearing LLR [12]. Varus was handled as negative values, whereas valgus was handled as positive values. The posterior tibial slope (PTS) was measured preoperatively, and at 4.5 and 12 months postoperatively, using conventional lateral knee radiographs. PTS was measured as the angle between a tangent to the medial tibial plateau and a line connecting the middle of the proximal tibia at 5 and 15 cm distal to the knee joint [23]. Postoperative evaluation for the event of hinge‐fractures was performed using intraoperative fluoroscopic images, as well as using conventional radiographs and CT at 4.5 months postoperatively. Hinge‐fracture classification was performed according to the Takeuchi classification [21].

### Statistical analysis

Sociodemographic and clinical characteristics of patients were determined using descriptive statistics. Continuous variables are reported as mean and standard deviation (SD). Normality of distribution was tested using the Shapiro–Wilk test. Accordingly, the two‐tailed paired *t*‐test or Wilcoxon test was applied to assess differences between pre‐ and postoperative parameters. Surgical accuracy of HKA and PTS correction was calculated as absolute values of the difference of reached correction—planned correction. All statistical analyses were performed in SPSS for Windows (Version 29.0, SPSS Inc.). Statistical significance was set at *p* < 0.05.

## RESULTS

Fourteen knees (5 right side, 9 left side) in 14 patients (6 females, 8 males) were included in this study. Mean age at time of surgery was 44.4 ± 9.9 years (range 18.0–55.0 years). Mean body mass index (BMI) was 29.1 ± 5.5 kg/m² (range: 20.4–38.0 kg/m²). Mean surgery time was 54.6 ± 15.8 min (range: 34.0–86.0 min). Overall, 2 hinge fractures (14.3%) were detected, including 1 Takeuchi I and 1 Takeuchi III hinge fracture. The Takeuchi III hinge fracture was only visible in the CT at 4.5 months postoperatively and healed without further treatment. Overall, no major intra‐ or postoperative complications, requiring surgical intervention, were observed during the follow‐up period. Pre‐ and postoperative HKA and PTS measurements are summarized in Table 1. Planned and reached HKA and PTS corrections (postoperative values–preoperative values) are summarized in Table 2. Accuracy for HKA correction was 1.9 ± 1.6° (range: 0.3–5.2°) at 4.5 months postoperatively and 1.9 ± 1.4° (range: 0.0–4.7°) at 12 months postoperatively. In all but one cases, the goal was to maintain the preoperative PTS. In one case, a correction of the PTS by −4.5° was aimed and the reached accuracy in this case was 2.3° at 4.5 and 12 months postoperatively. The overall accuracy for PTS correction was 0.8 ± 1.0° (range: 0.0–3.5°) at 4.5 months postoperatively and 1.0 ± 0.9° (range: 0.0–3.4°) at 12 months postoperatively. The knee society score (KSS) [7] was collected preoperatively and at a mean of 14.7 ± 3.9 months (range: 11.6–22.9 months) postoperatively. It showed a significant improvement for the clinical score from 57.4 ± 19.4 (range: 27.0–90.0) to 84.1 ± 16.6 (range: 42.0–100.0) (*p* = 0.003) and for the function score from 80.4 ± 18.4 (range: 45.0–100.0) to 93.6 ± 11.5 (range: 60.0–100.0) (*p* = 0.043).

**Table 1:** Pre‐ and postoperative hip‐knee‐ankle angle (HKA) and posterior tibial slope (PTS) measurements.

	Preoperatively	4.5 months postoperatively	12 months postoperatively
HKA (°)	−6.7 ± 1.9 (range: −11.7 to −4.0)	−1.9 ± 2.4 (range: −6.7 to 1.9)	−1.8 ± 2.1 (range: −6.0 to 0.9)
PTS (°)	9.3 ± 3.4 (range: 0.6 to 13.5)	9.5 ± 2.6 (range: 4.1 to 14.5)	9.6 ± 2.7 (range: 4.0 to 14.5)

Abbreviations: HKA, hip‐knee‐ankle anlge; PTS, posterior tibial slope.

**Table 2:** Planned and reached hip‐knee‐ankle angle (HKA) correction and posterior tibial slope (PTS) correction at 4.5 and 12 months postoperatively.

	Planned	Correction at 4.5 months postoperatively	Correction at 12 months postoperatively	*p* value
HKA (°)	6.6 ± 1.1 (range: 5.0 to 8.0)	4.8 ± 1.9 (range: 0.8 to 8.4)	4.9 ± 1.8 (range: 1.3 to 7.4)	0.002/0.002
PTS (°)	−0.3 ± 1.2 (range: −4.5 to 0.0)	0.3 ± 1.2 (range: −2.2 to 3.5)	0.3 ± 1.3 (range: −2.2 to 3.4)	0.054/0.064

Abbreviations: HKA, hip‐knee‐ankle angle; PTS, posterior tibial slope.

## DISCUSSION

The most important finding of this study is that the use of the here investigated personalized HTO system, using PSI and PSP, results in accurate accuracy of HKA and PTS correction as well as significant improved patient‐reported outcome measures after 1‐year follow‐up. Hence, the hypothesis of this study could be confirmed.

Especially in younger adults, HTO is preferred over arthroplasty in case of an unbalanced load distribution in the knee caused by a varus malalignment of the HKA [19]. It was shown that patients after HTO show better range of motion, a faster return to sports activities, and overall, a higher postoperative level of activities, compared to patients undergoing UKA [8, 18, 25]. Long‐term follow‐up of 20 years showed a survival rate of nearly two‐thirds in favourable candidates with a high satisfaction rate of 97% [3]. However, accurate correction of the HKA is a key prerequisite in performing HTO. Different studies showed that under‐ or overcorrection in HTO are main reasons for clinical failure with inferior patient‐reported outcome measures [2, 15]. An insufficient correction of the HKA also bears the risk of a recurrence of a varus HKA [20]. Various efforts have been made to enhance surgical accuracy in HTO, including the introduction of PSI. PSI enable a comprehensive 3D preoperative planning for deformity correction and seamless implementation during surgery, but require a preoperative CT scan, and therefore, the additional radiation should be taken into account. This technique has been widely and successfully utilized in knee arthroplasty [17, 22], and the advantages have also been demonstrated in joint‐preserving deformity correction across various anatomical locations [10, 13, 14]. Different studies investigated the accuracy of PSI in HTO, and accuracy for HKA correction ranges between 0.8° and 2.1°, whereas accuracy for PTS correction ranges between 0.2° and 2.7° [5, 6, 9, 24]. While PSI allow an enhancement of surgical accuracy, their capabilities have limitations at one point, driving the pursuit of additional tools to achieve even greater surgical accuracy. One such effort is the development of combined systems of PSI and PSP. By not only individualizing the surgical instruments but also customizing the used implants, further improvement of surgical accuracy may be achieved. A recent study by Zaffagnini et al. investigated the combined use of PSI and PSP for HTO in 25 patients using the TOKA (Tailored Osteotomy Knee Alignment) system (Orthoscape, UK) [24]. The PSP is specifically designed with a contoured undersurface that matches the individual tibial surface geometry. At 6 months postoperatively, they showed a surgical accuracy for HKA and PTS correction of 2.1 ± 2.0° (range: −1.1 to 5.8°) and 0.2 ± 0.4° (range: −0.6 to 1.2°), respectively. Considering these results, they are comparable to the results reported in the here presented study, with a surgical accuracy for HKA correction of 1.9 ± 1.4° (range: 0.0 to 4.7°) and a surgical accuracy for PTS correction of 1.0 ± 0.9° (range: 0.0 to 3.4°) at 12 months postoperatively. The main difference between the used PSI and PSP system of Zaffagnini et al. [24] and the here presented system is, that in the here presented system, a customized wedge is integrated in the undersurface of the plate, matching the required osteotomy gap. This may contribute to the slightly improved accuracy of HKA correction observed in this study. Overall, there is likely still room for improvement for combined PSI and PSP systems in HTO, as the current accuracy does not yet surpass that of PSI systems with traditional fixation plates. One possible reason is that traditional plates have been in use for many years, and surgeons are more familiar with these implants. Increased routine and familiarity with PSP may further enhance surgical accuracy over time. This is likely reflected in this study, as the case with the worst accuracy for HKA correction (4.7° at 1‐year follow‐up) was the only instance where the plate was improperly positioned, with the customized wedge placed outside the osteotomy gap. This case showed a postoperative HKA of −6.7° at 4.5 months and −6.0° at 1‐year follow‐up, representing an inacceptable correction in HTO. However, such inaccuracies may be avoided with increased familiarity with the implants, thereby enhancing surgical accuracy as experience grows.

Furthermore, in the here presented cases, surgery time varied widely. It can likewise be expected, that with increased familiarity, surgery time will be shorter and more stable.

This study should be interpreted in light of its potential limitations. First, only one reader performed the radiological measurements of HKA and PTS. However, high interrater reliability is already known for the assessed parameters [5, 12]. Secondly, the retrospective character of this study has to be considered, but this should probably not impair the report of the surgical accuracy of this technique. Finally, the limited number of included patients should be considered, and a larger cohort would be favourable. However, literature on combined PSI and PSP systems is very limited, making even a small cohort valuable for current research.

## CONCLUSION

The investigated combined PSI and PSP system shows accurate surgical accuracy for HKA and PTS correction with significant improvement of patient‐reported outcomes at 1‐year follow‐up.

## AUTHOR CONTRIBUTIONS

All authors have made substantial contributions to all of the following: (1) the conception and design of the study, or acquisition of data, or analysis and interpretation of data, (2) drafting the article or revising it critically for important intellectual content, (3) final approval of the version to be submitted.

## CONFLICT OF INTEREST STATEMENT

Sandro F. Fucentese is a consultant of Medacta (Medacta, Castel San Pietro, Switzerland).

## ETHICS STATEMENT

The local ethical committee approved this retrospective study (Zurich Cantonal Ethics Commission, BASEC‐Nr. 2023‐00389). Informed consent was obtained from all study participants.

## Data Availability

The data that support the findings of this study are available from the corresponding author upon reasonable request.
